# Diagnosis and treatment of gastric abscess by endoscopic ultrasound: A mini‐review of the preliminary application

**DOI:** 10.1002/deo2.70129

**Published:** 2025-05-07

**Authors:** Jia Xie, Mo‐Jin Wang, Rui Wang

**Affiliations:** ^1^ Department of Gastroenterology West China Hospital of Sichuan University Chengdu China; ^2^ Gastric Cancer Center West China Hospital of Sichuan University Chengdu China

**Keywords:** drainage, endoscopic submucosal dissection, endoscopic ultrasound, gastrectomy, gastric abscess

## Abstract

Gastric abscess is a rare condition caused by gastric barrier damage. It is easily misdiagnosed in clinical practice as a cancer recurrence or submucosal tumor, especially after surgery or endoscopic submucosal dissection. With a relatively high mortality rate, the cause and clinical characteristics of gastric abscesses are obscure. To date, diagnostic evaluations have mostly included indirect gastroscopy and abdominal computed tomography. A definite diagnosis of gastric abscess is challenging, and unnecessary surgery is sometimes performed. Relatively few applications of endoscopic ultrasound (EUS) have been described. EUS‐guided fine needle aspiration for diagnosis and drainage is not commonly used. Therefore, more experiences related to the cause and clinical characteristics of gastric abscesses should be reported. Further recognition of EUS ultrasonographic images and related minimally invasive EUS therapies are urgently needed. Herein, through a literature review of previous cases, we summarized the causes, clinical features, and diagnostic methods for gastric abscess. Moreover, we aimed to gain more experience diagnosing gastric abscesses by EUS for future differentiation and treatment strategies by endoscopy.

## INTRODUCTION

A gastric abscess is a localized, pyogenic, purulent abscess of the gastric wall, and is one variety of phlegmonous or suppurative gastritis. It is usually characterized by the formation of a pocket of pus in the stomach wall. The localized gastric abscess accounts for 5%–15% of all suppurative gastritis.^1^ Gastric abscess usually occurs due to gastric barrier damage. As a rare complication, gastric abscess after surgery or endoscopic submucosal dissection (ESD) has been reported in only a few cases.^2^, ^3^ However, it is difficult to differentiate it from cancer recurrence and submucosal tumors (SMTs). To date, diagnostic evaluations have mostly included indirect gastroscopy and abdominal computer tomography (CT). A definite diagnosis of the gastric wall is still challenging, and unnecessary surgery is sometimes performed.^3^ Relatively few applications of endoscopic ultrasound (EUS) have been described. EUS‐guided fine needle aspiration (EUS‐FNA) for diagnosis and drainage is rare.^4^, ^5^, ^6^ Therefore, more experiences related to the clinical characteristics of gastric abscesses should be shared. Further recognition of EUS ultrasonographic images and related minimally invasive therapies are urgently needed. Herein, through a literature review of previous cases, we summarized the clinical features and diagnostic methods for gastric abscess. Moreover, we aimed to gain more experience with EUS manifestations for future differentiation and treatment strategies by endoscopy and EUS.

### Methodology

We performed a systematic search of the English literature published in the PubMed, Embase, and Cochrane Library databases using the following keywords: “gastric abscess”, “endoscopic ultrasound”, “endosonography”, “endoscopy”, “fine needle aspiration”, “fine needle biopsy”, “interventional EUS”, “gastrectomy”, “endoscopic submucosal dissection”, “drainage”, “endoscopic drainage”. All the authors participated in the search and selection of relevant studies.

### Cause of gastric abscess

The causes of gastric abscesses are obscure. It usually occurs due to gastric barrier damage, originating from gastritis, ulcers, perforation, cancer, stromal tumors, large polyps, foreign bodies, etc., on the basis of which pathogenic bacteria reproduce, leading to acute suppurative inflammation. Infection from other adjacent areas, such as acute cholecystitis, biliary tract infection, acute pancreatitis, and liver abscess, might also invade the gastric wall. Hematogenous spreading by distant infection metastasis was also observed (Table 1).^7^ There was currently no evidence of Helicobacter pylori infection, drugs (such as steroids or nonsteroidal anti‐inflammatory drugs), or proton pump inhibitors related to the risk of gastric abscess.

**TABLE 1:** Clinical characteristics of gastric abscess.

Author	Gender	Age	Complications	Procedures	Occurrence time	Clinical manifestation	Gastric location	Size (cm)	Imaging to diagnosis	Morphology under endoscopy	Treatments	Follow‐up
Kiil et al., 2001^8^	Male	63	Bleeding esophagitis	/	/	/	Antrum	3×3×3	EGD	Submucosal mass with ulcer and pus	Endoscopic drainage	C, CT, EGD
Choong et al., 2003^9^	Male	75	Supranuclear palsy, idiopathic dilated cardiomyopathy	Cause by fish bone	**/**	Epigastric pain, fever, chills	Antrum	4×3	CT, EUS+FNA, cultures	Submucosal mass	Antibiotics, drainage	C, CT
Huang, 2003^10^	Male	45	/	/	/	Abdominal pain, fever	Anterior wall	/	CT, EGD	Submucosal mass, thickening of gastric folds	Endoscopic drainage, antibiotics, abstinence from food	C
Marcos et al., 2010^11^	Male	48	Hiatal hernia, erosive gastritis, gastroesophageal reflux	**/**	**/**	Epigastric pain, fever	Large curvature near the antrum	10	abdominal ultrasound, CT, EUS, cultures	Well‐circumscribed intramural mass	Endoscopic drainage	C, EUS
Dohi et al., 2014^12^	Female	63	/	ESD	5th‐day post‐ESD	Abdominal pain, vomiting	Body	/	CT, EGD	Fistula at the edge of post‐ESD ulcer, pus flow	Endoscopic drainage	C, CT
Mandai et al., 2016^13^	Male	84	/	/	/	Tarry stools	Posterior wall	/	EGD, EUS	Submucosal tumor, erosion, and white coat on top	EUS‐guided drainage	C, CT
Kobayashi et al., 2016^14^	Female	75	Pancreatic tumor with liver metastases, diabetes mellitus	EUS‐FNA	2 weeks	Abdominal pain, fever	Posterior wall	3.5	CT, EGD, EUS	Submucosal lesion	Antibiotic	C, CT
Mufty et al., 2017^15^	Female	59	/	Mesenteric bypass surgery	15 years	Fever, urinary frequency, lower backache	Mass around stomach	/	CT	Thick wall with erosion	Surgery	C
Movahed et al., 2017^16^	Male	70	Morbid obesity	Laparoscopic adjustable gastric banding	4 years	Malaise, abdominal pain, poor appetite, fever	Anterior wall	3–4	CT, EUS+FNA, culture	Large obstructive bulge	EUS+ fluoroscopic guided drainage	Surgery
Wu et al., 2018^17^	Male	55	Pancreatic adenocarcinoma	EUS‐FNA for pancreatic mass	1 week	Upper abdominal pain, fever	Posterior wall	/	CT, EGD	mass	Antibiotic, endoscopic drainage	C, CT
Chen et al., 2018^18^	Female	47	/	A gastric wall abscess involving the colon	/	Abdominal pain	/	/	EGD, CT, EUS	/	surgery	C
	Male	31	/	Metastasized gastric cancer from the colon	/	Abdominal pain	/	/	EGD, CT, EUS	/	Surgery	Died
Kimura et al., 2019^19^	Female	68	Pancreatic tail adenocarcinoma, diabetes mellitus	EUS‐FNA for pancreatic mass	1month	Abdominal pain, fever	Posterior wall	4.4	CT, EGD, EUS, culture	A bulging mass with purulent fluid	Endoscopic drainage	C, CT
Asayama et al., 2021^5^	Male	72	/	ESD	28 days	Epigastric pain	Greater curvature	6	CT, EGD	Submucosal tumor around post‐ESD ulcer	EUS‐FNA	C, CT
Yu et al., 2021^20^	Female	72	/	ESD	10 weeks	/	Posterior wall of the antrum	5	CT, EGD	subepithelial lesion	Gastrectomy	C
Takayanagi et al., 2022^21^	Male	78	Esd delayed perforation	ESD	5 days	Abdominal discomfort, fever	Lesser curvature	5	CT, EUS	Fistula on abscess under EUS	Antibiotics, proton pump inhibitor, parenteral nutrition+ EUS/fluoroscopic‐guided drainage	C, CT
Qafisheh et al., 2022^22^	Female	50	Hypertension, asthma	/	/	Epigastric pain, nausea, vomiting, passing blood with defecation	prepyloric area	/	Abdominal ultrasound, CT, EGD EUS, pathology	Mass‐like lesion	EUS+FNA+ drainage, antibiotics	C
Ogino et al., 2022^23^	Female	77	Advanced gastric cancer	/	/	Epigastric pain	Greater curvature of the body	7	CT, EGD, EUS	mass	Endoscopic drainage, distal gastrectomy, Roux‐en Y gastrojejunostomy	C
Kim et al., 2023^24^	Male	75	Calculous cholecystitis	/	/	Epigastric pain, anorexia, vomiting	Antrum	4	CT, EGD, EUS	subepithelial tumor	Endoscopic drainage	C

Abbreviations: C, clinical recovery; CT, computed tomography; EGD, esophagogastroduodenal endoscopy; EUS, endoscopic ultrasound.

**FIGURE 1:**
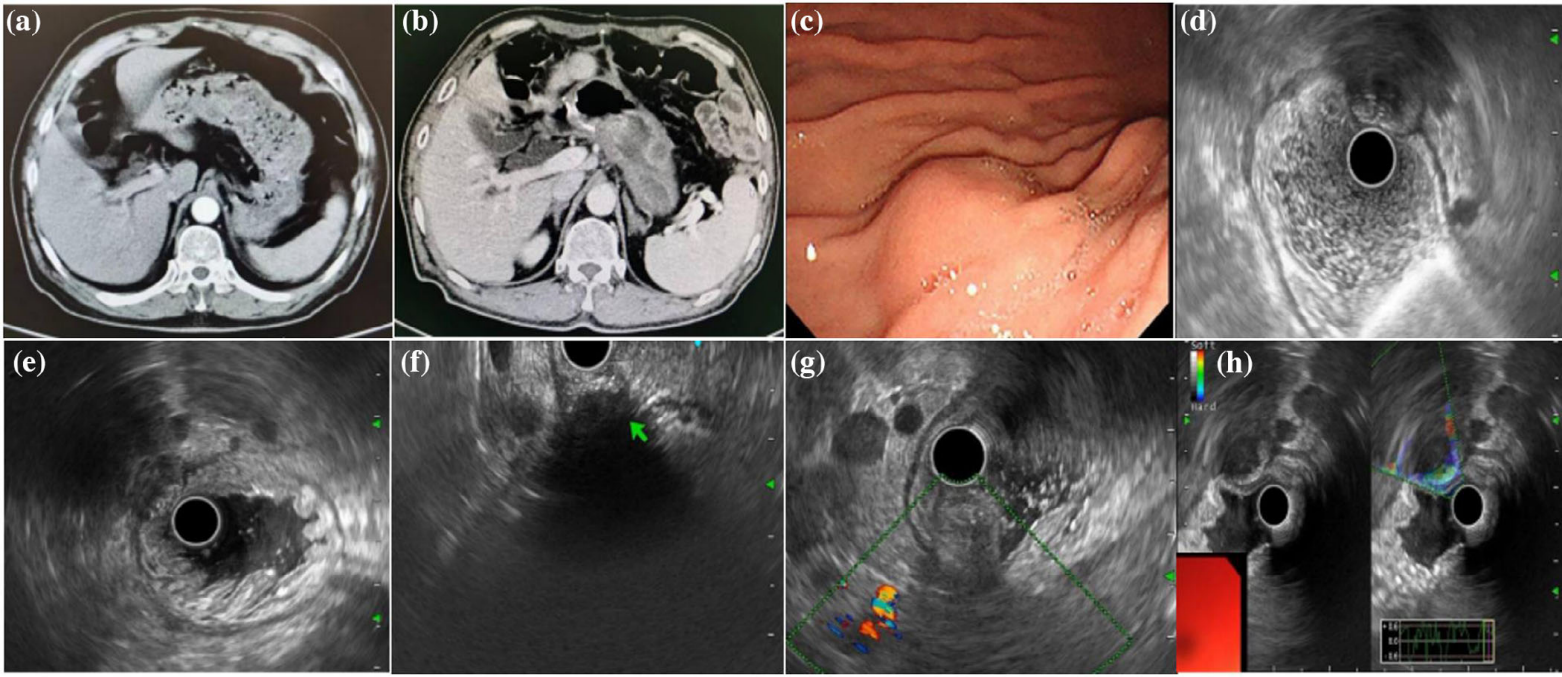
A case of gastric abscess from the Department of Gastroenterology, West China Hospital. (a) No nodule in enhanced computed tomography before surgery. (b) A newly emerged low‐density mass distant from the original surgical site in the submucosa of the gastric body. (c) Gastroscopy showed a hemispherical protuberant lesion appearing as a submucosal tumor. (d–f) 20 MHz probe, radial, and linear endoscopic ultrasound showed an oval, mixed hyperechoic and hypoechoic mass suspiciously originating from the submucosa or muscularis propria layer of the gastric wall. The endoscopic submucosal dissection boundary of mass was obscure and the serosa layer was unclear due to the influence of hyperechoic sound shadow. (g) There was no obvious blood flow inside the lesion under Doppler mode. (h) Mass was soft under Elastography mode. An area with nearly an echo and no signal for elastography indicated liquefaction inside.

Table 1 shows the clinical characteristics of the patients with gastric abscesses.^5^, ^8^, ^9^, ^10^, ^11^, ^12^, ^13^, ^14^, ^15^, ^16^, ^17^, ^18^, ^19^, ^20^, ^21^, ^22^, ^23^, ^24^ Abscesses after invasive gastric procedures for treatment like surgery or ESD should not be ignored. As a rare complication, gastric abscess after surgery or ESD has been reported in only a few cases.^2^, ^3^ Reports have shown that gastric surgery and ESD might be high‐risk factors for gastric abscesses, as the same in the case of our institution (Figure 1A, B). A 67‐year‐old male presenting a gastric abscess away from the surgical anastomosis 10 months after radical distal gastrectomy for gastric adenocarcinoma. Gastric abscesses occur approximately 5 days to 3 months after ESD, usually due to ulcers, fistulas, or perforation.^5^, ^12^, ^20^, ^21^ Gastric abscesses after surgery, including mesenteric bypass surgery and laparoscopic adjustable gastric banding, occur at 15 and 4 years.^15^, ^16^

The possibility of gastric abscess after EUS‐FNA for pancreatic cancer should be considered. A 72‐year‐old woman complained of fever 18 days after EUS‐FNA for pancreatic ductal adenocarcinoma of the pancreatic tail. An abscess associated with a pancreatic fistula containing necrotic debris formed in the EUS‐FNA needle tract.^25^ There were three other cases of gastric abscess after EUS‐FNA of the pancreatic tumors. The abscesses are located at the posterior wall within one week to one month, where they can cause abdominal pain and fever.^14^, ^17^, ^19^ Three patients were elderly, and two had diabetes mellitus. Therefore, endoscopists should be vigilant of gastric abscess formation after FNA in immunocompromised patients with recurrent symptoms. Fortunately, those abscesses were smaller than 4.5 cm and were successfully treated with endoscopic drainage and/or antibiotics.

### Clinical characteristics of patients with gastric abscess

Of the 19 patients with gastric abscesses reported, 11 were male and eight were female. The mean age was 63.5 years, and 63.2% were older than 60 years. Patients often presented nonspecific clinical manifestations. In terms of symptoms recorded, abdominal pain/discomfort, fever, and nausea/vomiting were most common, accounting for 88.2% (15/17), 52.9% (9/17), and 17.6% (3/17) of the patients, respectively (Table 1). Patients sometimes lose their appetite (2/17). Florid signs of a septic focus were absent. Therefore, gastric abscesses could be neglected by patients and missed by doctors. Based on limited records, the diagnostic time interval from initial symptoms fluctuated from 5th‐day post‐ESD to 15 years after mesenteric bypass surgery. 66.7% (6/9) of patients presented as an acute process within 1 month. From available records of blood examination, 81.2% (13/16) and 81.8% (9/11) patients showed an elevated level of white blood cell count and C‐reactive protein.

### Differentiating gastric abscess from malignancies

Several imaging techniques are helpful for identifying intramural gastric abscesses. Nevertheless, a definite diagnosis is still challenging. The difficulty exists in differentiating it from cancer recurrence and SMTs. 27%–32.4% of patients in China suffer from local‐regional recurrence at the remnant stomach or lymph node within 11–36 months after surgery.^26^, ^27^ Compared with patients who underwent surgery, patients who underwent ESD had even greater risks of local recurrence.^28^ Therefore, it is essential to differentiate benign from malignant masses found around the stomach; otherwise, unnecessary surgery should be performed.^3^ Chen et al. first reported two cases of gastric wall abscesses that were ultimately diagnosed by surgery with suspicion of cancer metastasis and suggested multiple methods including gastroscopy, abdominal CT, and EUS ± FNA, as part of the routine examination.^18^

### Imaging for gastric abscess

To date, diagnostic evaluations have mostly included indirect imaging via abdominal CT, gastroscopy, and EUS. Abdominal‐enhanced CT was performed in 89.5% (17/19) of patients and was regarded as the first choice for early diagnosis (Table 1). CT usually shows stomach wall thickening with a mass‐like lesion with uneven enhancement. It can present as solid lesions or cystic‐solid lesions with blurred margins, and there is sometimes gas in some lesions. Occasionally, there was interference from the punctate high‐density shadows inside the lesion, which might be initially diagnosed as a mixed infection of tuberculosis and inflammatory exudation. High‐density shadows might have formed because of the foreign body, which contained bone or other high‐density substances and was partially encapsulated by inflammatory tissue to form an abscess. Enhanced CT allowed rapid diagnosis and demonstrated the location, size, and extent but not the specific etiology of the lesion. Especially, it was difficult to distinguish the originating layer from the gastric wall, and this layer may be confused with the SMT.

Gastroscopy was the second most common method for measuring gastric abscesses in 73.7% (14/19) of patients. Under gastroscopic guidance, the location of the gastric abscesses was flexible. It is usually located at the gastric antrum or body, either at the anterior or posterior wall or at the lesser or greater curvature. It presented as a submucosal mass or thickening of the gastric folds. It can reportedly appear similar to an SMT (Figure 1C). Ulcer, fistula, and erosion may occasionally appear above the lesion in five patients.^8^, ^12^, ^13^, ^15^, ^21^ The diagnosis could be definitively established if pus is covered or leaked from the lesion. However, it cannot usually be captured during endoscopy.

### Use of EUS for gastric abscesses

Because of the complexity of the diagnosis, multiple methods are needed. EUS ± FNA was combined with CT and/or gastroscopy in 36.8% (7/19) of patients. High‐frequency EUS ± FNA greatly improved the accuracy of diagnosing gastric abscesses. Moreover, EUS+FNA provides an opportunity to arbitrate infection and malignant or benign tumors to identify specific pathogens and, in cases of localized gastric abscesses, for resolution by decompression. More advanced endoscopic procedures have rapidly emerged to supplement EUS+FNA, which has already been demonstrated to improve minimally invasive diagnosis and to be effective management for gastric abscesses.

Seven patients (36.8%) underwent EUS for gastric abscess detection as shown in Table 2.^9^, ^11^, ^14^, ^18^, ^19^, ^24^, ^29^ EUS+FNA/culture for diagnosis was employed in 4 patients, which is even less commonly used.^9^, ^11^, ^16^, ^22^ Therefore, further recognition of EUS ultrasonographic images and related minimally invasive therapies by endoscopy are urgently needed.

**TABLE 2:** Characteristics of gastric abscess under endoscopic ultrasound.

Author	EUS	Size	Shape	Location	Echo intensity	Homogeneity	Blood flow	Elasticity	Involved hierarchical structure
Choong et al., 2003^9^	Radial and linear EUS	4 × 3 cm	Well‐circumscribed intramural mass	Antrum	The presence of internal fluid and debris	Mixed echogenicity, hyperechoic linear structure inside#	/	/	/
Tsai et al., 2008^29^	mini probe (12 MHz)	3.7 × 6.6 cm	Well‐circumscribed mass	Posterior wall	Hypoechogenicity	/	/	/	Fourth layer
Marcos et al., 2010^11^	Linear EUS	10 cm	Well‐circumscribed intramural mass	Large curvature near the antrum	The presence of internal fluid and debris	Mixed echogenicity	/	/	/
Kobayashi et al., 2016^14^	/	3.5 cm	Well‐circumscribed mass	Posterior wall	/	Mixed echogenicity	/	/	/
Chen et al., 2018^18^	/	3 × 5 cm	/	/	Hyperechogenicity	/	/	/	Fourth layer
Kimura et al., 2019^19^	Linear EUS	4.4 cm	/	Posterior wall	Hyperechogenicity	Mixed echogenicity	/	/	Second to the fourth layer
Kim et al., 2023^24^	/	4.3 × 2.5 m	Mass	Anterior wall of the antrum	Filled with liquified material, and air‐fluid level	Mixed echogenicity	/	/	Third layer

#: Fish bone inside.

### Characteristics image of the gastric abscess under EUS

There are relatively few applications of EUS for diagnosis, as shown in Table 2 (Figure 1D–F).^9^, ^11^, ^14^, ^18^, ^19^, ^24^, ^29^ Linear and radial EUS methods other than a mini probe are usually used to visualize the whole mass because it is often larger than 3–10 cm. A well‐circumscribed, circular intramural mass, anechoic, hypo‐ or hyper‐echogenicity, mixed echogenicity with internal fluid and debris, and usually a second (submucosa) to fourth layer (muscularis propria) involvement under EUS might suggest an abscess. It could be different from cancer recurrence or metastasis (irregular shape, hypoechoic, first to fourth layer) and SMT (well‐circumscribed, hypoechoic, second or fourth layer). The presence of internal fluid and debris or air‐fluid levels might be strong reminders for infection and benign lesions. However, there are also very confusing situations. Cancer recurrence or metastasis into the stomach wall may become necrotic, therefore, anechoic‐hypoechoic lesions with mixed echogenicity and invasion into the second layer (submucosa) to the fourth layer (muscularis propria) could also be observed. In such cases, it is indistinguishable from an intra‐gastric abscess. Previous cancerous history and elevated tumor markers may offer the clue. EUS‐FNA can provide differentiation pathology although at a relatively low risk of possible needle tract seeding about 0.003%–0.009%. There was no information from previous reports of gastric abscess under Doppler mode or elastography mode. One patient showed no obvious blood flow in the lesion under Doppler imaging. The mass was soft under elastography. An area with nearly an echo and no signal for elastography indicated liquefaction inside (Figure 1G,H). Doppler or elastography might provide guidance for accurate differential diagnosis in the future. It might be used as a future exploration to distinguish between gastric abscesses and necrotic metastatic tumors.

### Diagnostic value of EUS‐FNA for gastric abscess

**FIGURE 2:**
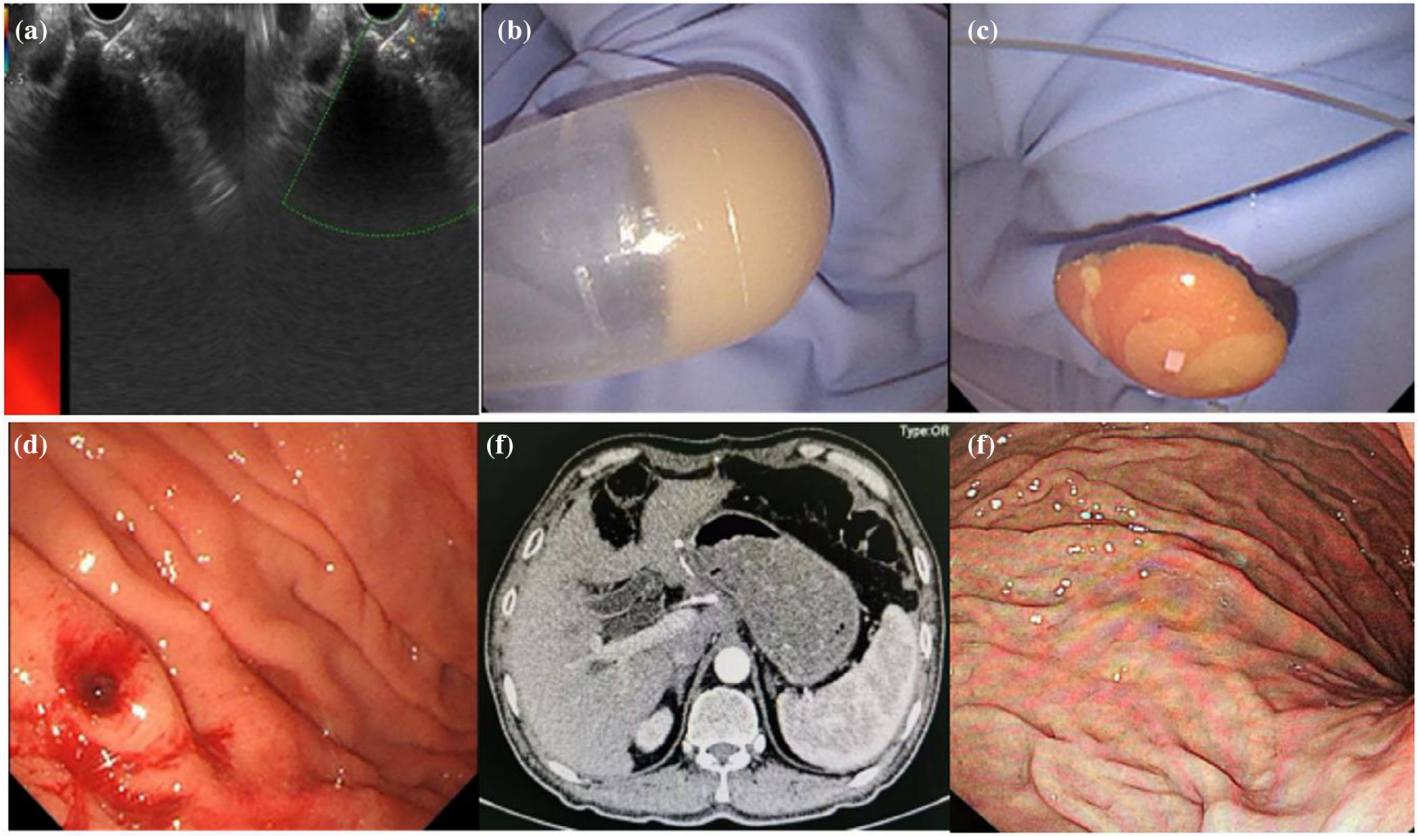
(a) Endoscopic ultrasound‐guided aspiration by a 22G puncture needle was performed. (b, c) Obtained specimens showed yellow purulent fluid with broken tissue. (d) After aspiration, the lesion was flattened without active bleeding. (e, f) Six months later, abdominal computed tomography and gastroscopy showed gastric nodule disappeared.

EUS‐FNA or deep forceps bites can be used to obtain samples of gastric abscess pathogens. Even when the culture failed, pus aspirated by EUS+FNA or forceps bite was a direct indicator (Figure 2A–C). Although the pathogens causing gastric abscesses are diverse, most of these infections involve infections by the oral microflora. Streptococci are the most common pathogens isolated from gastric abscess cultures, accounting for approximately 70% of cases, and a variety of aerobic and anaerobic bacteria, including *Escherichia coli*, *Proteus vulgaris*, *Clostridium perfringens*, *Clostridium welchi*, Pseudomonas aeruginosa, *Proteus mirabilis*, *Staphylococcus* and *Bacillus subtilis*, as well as various fungi, have been identified.^7^ EUS+FNA culture aided in the selection of appropriate antibiotics for the pathogen. Cephalosporins and quinolones are usually present.

Caution should be taken when determining whether gastric abscesses can be caused by metastatic gastric cancer or advanced gastric cancer perforation. FNA for cytology or close follow‐up was beneficial for this purpose to avoid missing potential underlying malignancies.^18^, ^23^ A newly emerged mass distant from the original surgical site was even worthy of EUS+FNA for biopsy, especially when in situ recurrence was not considered. EUS+FNA for biopsy was advantageous over CT alone.

### Conventional treatment for gastric abscess

A high mortality rate of patients with suppurative gastritis, between 37% and 84%, has been reported.^7^, ^30^, ^31^, ^32^ Even after accurate diagnosis, treatments for gastric abscess were previously restricted to surgical resection in combination with antibiotics. Miller et al. reported a mortality rate of 100% in patients treated medically, compared with 18% in patients treated with gastric resection and antibiotics.^33^ With the update of treatment concepts, only 26.3% (5/19) of patients underwent surgery (Table 1), with two patients having cancer as the foundation.^18^, ^23^ Ogino et al. reported that a patient whose condition did not improve after endoscopic drainage in combination with antibiotics underwent distal gastrectomy with D2 lymphadenectomy; fortunately, the tumor with the abscess was safely and curatively removed without perforation.^23^ These findings indicate that patients with recurrent symptoms or who are difficult to drain should be cautious of accompanying malignancies and sometimes need surgical treatment.

### Drainage under EUS for gastric abscess

Surgical procedures were conventionally used for abscess drainage, but there were drawbacks including high incidences of complications, costs, and long hospitalization. 26.3% (5/19) of gastric abscesses undertook surgery with clinical recovery in four cases, but one patient died afterward (Table 1). To avoid surgical complications, other therapeutic options, such as percutaneous drainage and endoscopic drainage, have emerged over time. Recently, therapeutic endoscopic drainage has become a promising method for treating intramural gastric abscesses for its minimal invasiveness. However, due to the difficulties in endoscopic technique and interdisciplinary collaboration, endoscopic drainage especially under EUS has not been carried out in multi‐centers. In total, 68.4% (13/19) of the gastric abscesses were relieved by endoscopic drainage in combination with antibiotics (Table 1). Endoscopic drainage reached a high clinical and technical success rate of 92.9%, except for one patient who turned to surgery for help because a gastric abscess occurred based on advanced gastric cancer.^23^ This finding suggested that endoscopic drainage has received increasing recognition and has become a first‐line strategy (Figure 2D–F).

The means of the endoscopic drainages are displayed in Table 3.^5^ **^,^** ^8^, ^9^, ^10^, ^11^, ^12^, ^13^, ^16^, ^17^, ^19^, ^21^, ^22^, ^23^, ^24^ In general, they are classified as internal or external drainages. Internal drainages included simple EUS‐FNA aspiration and opening window fistulotomy by simple forceps bites, rat‐toothed forceps, and a needle knife or hook knife to make a large hole, with or without pigtail catheter or stent insertion through the hole. ^8^, ^11^, ^13^, ^16^, ^17^ The hole could be opened up to 1.5–2.0 cm or further expanded by a balloon dilatation catheter on top of the mass to facilitate drainage.^5^, ^19^, ^24^ Saline irrigation into the cavity followed by aspiration was used for clearer lavage.^19^, ^24^ Four patients reported external drainages, including percutaneous drainage with a pigtail catheter or a nasocystic catheter. ^5^, ^9^, ^12^, ^21^ However, only one patient received external drainage alone.^21^ The other three combined internal and external drainages were used.

**TABLE 3:** Endoscopic treatments for gastric abscess.

Author	Endoscopic treatments
Kiil et al., 2001^8^	Forceps bite to drain
Huang et al., 2003^10^	Endoscopic drainage
Choong et al., 2003^9^	EUS+FNA + percutaneous drainage with a pigtail catheter
Marcos et al., 2010^11^	Endoscopic drainage with needle knife + pigtail catheter
Dohi et al., 2014^12^	Double pigtail stent internal + nasocystic catheter external drainage
Mandai et al., 2016^13^	EUS+FNA + needle knife + internal stents
Movahed et al., 2017^16^	EUS+FNA + fluoroscopic guidance double pigtail stent
Wu et al., 2018^17^	Endoscopic drainage by hook knife
Kimura et al., 2019^19^	Endoscopic unroofing drainage
Asayama et al., 2021^5^	EUS+FNA + double‐pigtail stent internal + nasobiliary tube external drainage
Qafisheh et al., 2022^22^	EUS+FNA + needle knife drainage
Takayanagi et al., 2022^21^	EUS+FNA + fluoroscopic guidance nasobiliary tube drainage
Ogino et al., 2022^23^	EUS‐guided drainage by needle knife, unsuccessful
Kim et al., 2023^24^	Endoscopic incision by H‐knife

Abbreviations: ERCP, endoscopic retrograde cholangiopancreatography; EUS, endoscopic ultrasound; FNA, fine needle aspiration.

In general, endoscopic drainage has been suggested to be effective and safe. There were seven cases of endoscopic drainage under EUS monitoring.^5^, ^9^, ^13^, ^16^, ^21^, ^22^, ^23^ All procedures were safely conducted without complications. EUS has the advantage of visualizing the drainage pathway to avoid blood vessels, thus providing safety assurance.

Only one case reported by Ogino et al. showed that endoscopic drainage was unsuccessful. The intramedullary abscess was rooted in an 82 × 65 mm long advanced gastric cancer lesion. It was formed from the ulcerative lesion of the cancer extending to the subserosa. EUS‐guided abscess drainage using a needle knife was performed first. However, it was very difficult to keep the endoscope view clear because of bleeding; thus, only a small amount of purulent discharge could be drained, which was insufficient even for bacterial culture. Therefore, the patient underwent distal gastrectomy with D2 lymph adenectomy, and fortunately, the tumor with the abscess was safely and curatively removed without perforation.^23^ The author indicated that abscess originating from the complex background of advanced gastric cancer with hemorrhage, necrosis, and thrombosis was the fundamental cause of endoscopic drainage failure. The choice of treatment plan still needs to be comprehensively evaluated based on diverse occasions.

### Timing of EUS for gastric abscess

**FIGURE 3:**
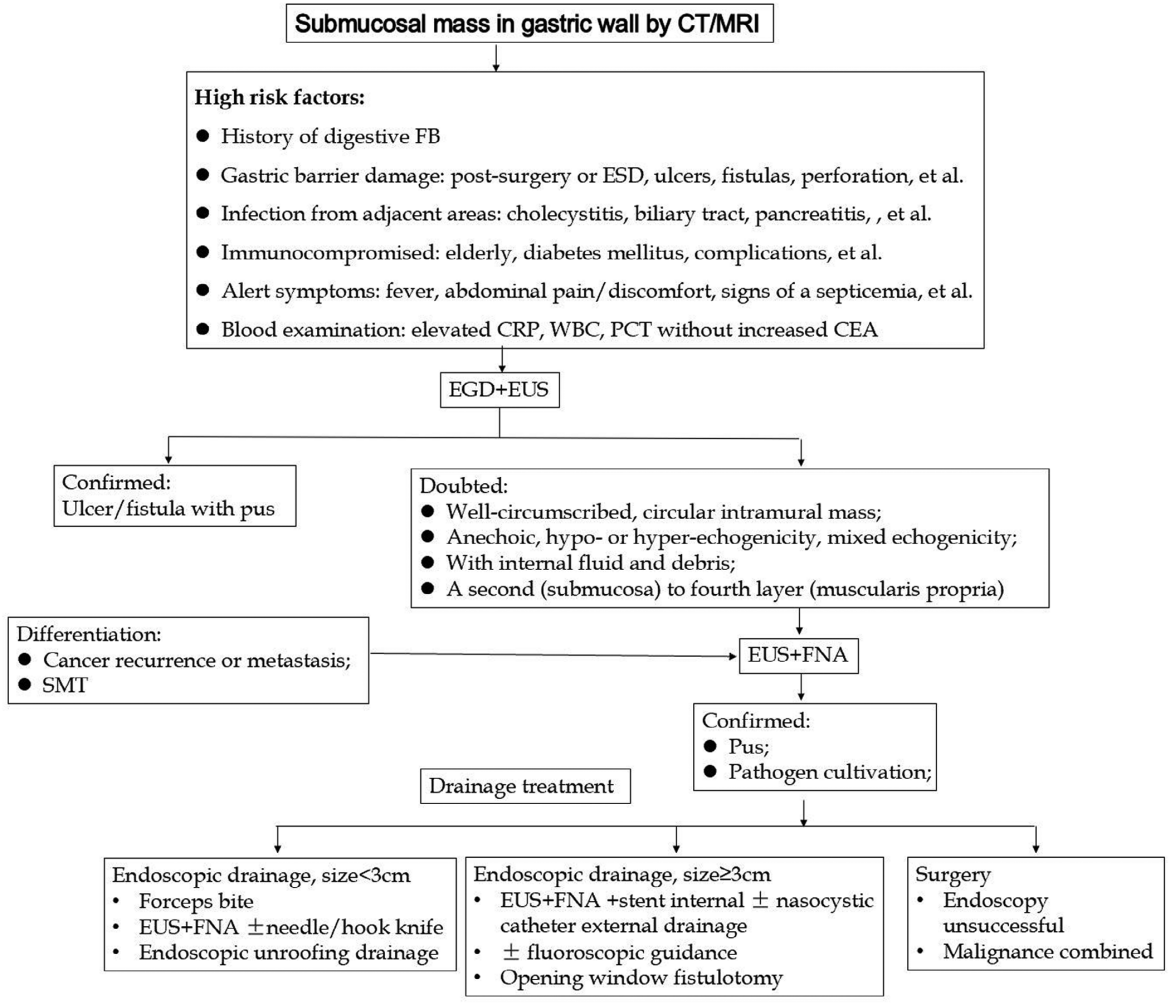
A diagnostic and drainage treatment flow‐chart for gastric abscess. CRP, C‐reactive protein; EGD, esophagogastroduodenal endoscopy; ESD, endoscopic submucosal dissection; CEA, carcinoembryonic antigen; FB, foreign body; FNA, fine needle aspiration; PCT, procalcitonin; SMT, submucosal tumors; WBC, white blood cell count.

To date, there is no consensus on endoscopic diagnosis and treatment especially under EUS for gastric abscess. We suggest that gastroscope and EUS ± FNA can be arranged for patients with high‐risk factors (Figure 3). Endoscopic strategies may depend on abscess size, location, endoscopic material, fluoroscopic guidance, etc., for full drainage of pus. Because of its viscous nature, sometimes combining internal and external drainage or surgery may be needed (Figure 3).^9^

## CONCLUSION

To summarize, gastric abscess, especially after surgery or ESD, is relatively rare and is easily misdiagnosed in clinical practice as cancer recurrence and SMT. A gastric abscess usually occurs in elderly patients after the gastric barrier is damaged. Patients presented with nonspecific abdominal pain and fever. A well‐circumscribed, circular intramural mass, hypo‐ or hyper‐echogenicity, mixed echogenicity with internal fluid and debris, and usually a second to fourth layer involved under EUS might suggest an abscess. EUS‐FNA can not only provide samples for pathogens but also rule out malignant lesions and SMTs and avoid unnecessary surgery. EUS+FNA‐based minimally invasive advanced endoscopic procedures were proven to be effective for full drainage of pus. We believe that with the increasing use of EUS, the mortality of patients with gastric abscesses will dramatically decrease.

## CONFLICT OF INTEREST STATEMENT

None.
